# Soziale Unterschiede im Gesundheitsverhalten verstehen und verändern: Ernährung als Schnittstelle zwischen sozialer Ungleichheit und Gesundheit

**DOI:** 10.1007/s00103-025-04099-1

**Published:** 2025-07-23

**Authors:** Núria Pedrós Barnils, Nourat Noemi Alazza, Christine Emmer, Carolin M. Callies, Jutta Mata, Benjamin Schüz

**Affiliations:** 1 Institut für Public Health und Pflegeforschung, Universität Bremen, Grazer Straße 4, 28359 Bremen, Deutschland; 2 Fakultät für Sozialwissenschaften, Lehrstuhl für Gesundheitspsychologie, Universität Mannheim, Mannheim, Deutschland; 3 Fakultät für Sozialwissenschaften, Mannheimer Zentrum für Europäische Sozialforschung, Universität Mannheim, Mannheim, Deutschland

**Keywords:** Ernährungsverhalten, Soziale Ungleichheit, Ernährungsumwelt, Einstellungen, Normen, Dietary behaviour, Social inequality, Food environment, Attitudes, Norms

## Abstract

Trotz eines gut ausgebauten Gesundheitssystems bestehen in Deutschland teils erhebliche soziale Unterschiede in der Gesundheit. Besonders sozial benachteiligte Menschen sind von höherer Krankheitslast und Mortalität betroffen. Ein großer Teil dieser Ungleichheiten lässt sich durch Unterschiede in gesundheitsrelevanten Verhaltensweisen wie Ernährung erklären.

Dieser Artikel beleuchtet theoretische Ansätze und aktuelle empirische Befunde aus der Perspektive von *Behavioural and Cultural Insights* – einer interdisziplinären Perspektive, die den Einfluss soziokultureller Faktoren auf Gesundheitsverhalten berücksichtigt –, um soziale Unterschiede in der Ernährung besser zu verstehen und gezielt zu verändern. Dabei spielen sowohl strukturelle als auch individuelle Faktoren eine wichtige Rolle: Die Ernährungsumgebung in sozial benachteiligten Regionen ist oft durch eine höhere Dichte von Fast-Food-Angeboten und mehr Werbung für ungesunde Nahrungsmittel geprägt. Gleichzeitig zeigen sich Unterschiede in verhaltensbezogenen Determinanten und deren Einfluss auf das Ernährungsverhalten.

Zur Verringerung sozialer Unterschiede in der Ernährungsqualität und damit verbundener gesundheitlicher Ungleichheiten sind Strategien sowohl auf struktureller als auch auf individueller Ebenen notwendig. Regulative Maßnahmen – etwa Werbebeschränkungen für ungesunde Lebensmittel, eine verbesserte Verfügbarkeit gesunder Angebote oder fiskalische Anreize – können die Ernährungsumgebung positiv verändern. Zeitgleich sind Interventionen nötig, die individuelle Fähigkeiten und soziale Ernährungssysteme stärken, beispielsweise durch frühzeitige Ernährungsbildung, gesündere Rahmenbedingungen in der Gemeinschaftsverpflegung oder Programme zur Förderung von Selbstwirksamkeit.

## Abstract (en)

Despite a well-developed healthcare system, significant social health disparities exist in Germany. People facing social disadvantages are particularly affected by a higher burden of disease and mortality. A large part of these inequalities can be explained by differences in health-related behaviours, in particular dietary behaviours.

This article examines theoretical approaches and current empirical findings through the lens of behavioural and cultural insights—an interdisciplinary perspective that considers the influence of sociocultural factors on health behaviours—to better understand and effectively address social differences in nutrition. Both structural and individual factors play a crucial role: in socially disadvantaged regions, the food environment is often characterized by a higher density of fast-food outlets and increased advertising for unhealthy foods. At the same time, differences in behavioural determinants and their influence on eating habits can be observed.

Reducing social disparities in diet quality—and the resulting health inequalities—requires strategies at both structural and individual levels. Regulatory measures, such as advertising restrictions on unhealthy foods, improved access to healthier options or fiscal incentives, can positively shape the food environment. At the same time, interventions are needed to strengthen individual skills and social food systems, for example through early nutrition education, healthier frameworks in communal catering or programs that promote self-efficacy.

Eigentlich ist es ein Unding: Auch in einem wohlhabenden Land wie Deutschland mit allgemeiner Krankenversicherung und einem großenteils gut funktionierenden Gesundheitssystem lassen sich eklatante soziale Ungleichheiten in der Lebenserwartung und in der Krankheitslast beobachten. Auswertungen des Sozio-oekonomischen Panels zeigen [1], dass die wohlhabendsten 10 % der Bevölkerung in Deutschland im Schnitt 7 Jahre länger leben als die ärmsten 10 %. Bei nichtübertragbaren Erkrankungen wie Krebs nehmen solche Ungleichheiten sogar zu: Während im Zeitverlauf die Anzahl von Krebsneuerkrankungen in der Gesamtbevölkerung in Deutschland zurückging, wurde der Unterschied zwischen benachteiligten und nicht benachteiligten Regionen über die Zeit größer [2] – der Rückgang bei Neuerkrankungen wurde nämlich vor allem in wohlhabenderen Regionen beobachtet. Ein erheblicher Teil dieser sozialen Unterschiede lässt sich auf eine gemeinsame Dimension zurückführen: soziale Unterschiede in gesundheitsrelevanten Verhaltensweisen [3]. Dabei nimmt – neben Rauchen – vor allem die Ernährung eine zentrale Stellung ein. Von allen sozialen Unterschieden in der Gesamtmortalität ließen sich in einer großen britischen Studie mit Angestellten des öffentlichen Dienstes bis zu 17 % durch Unterschiede in der Ernährung erklären [4]. In einer großen niederländischen Längsschnittstudie konnten sogar bis zu 67 % der bildungsbezogenen Unterschiede im Auftreten von Schlaganfällen durch Unterschiede im Ernährungsverhalten erklärt werden [5]. Soziale Unterschiede im Ernährungsverhalten lassen sich auf mehreren sozialen Dimensionen beobachten und sind nicht immer einheitlich. So zeigte sich beispielsweise in großen europäischen Untersuchungen, dass Teilnehmer:innen mit niedrigeren *Bildungsabschlüssen* mehr Fleisch und verarbeitetes Fleisch aßen [6], gleichzeitig konsumierten aber Teilnehmende mit geringeren *Einkommen* weniger verarbeitetes Fleisch als solche mit höheren Einkommen [7]. Wie sich solche sozialen Unterschiede im Ernährungsverhalten erklären und möglicherweise verändern lassen, ist weniger gut bekannt als das Ausmaß dieser Unterschiede – hier können *Behavioural and Cultural Insights* einen wichtigen Beitrag leisten. Dieser interdisziplinäre Ansatz bezieht den soziokulturellen Kontext mit ein, um Barrieren und förderliche Faktoren für Gesundheitsverhalten zu identifizieren und daraus evidenzbasierte, wirksame Interventionen abzuleiten.

Dabei ist allerdings sehr wichtig, dass die Ursachen für diese beobachteten sozialen Unterschiede im Ernährungsverhalten nicht allein *individuell* bei den Personen liegen, sondern durch *strukturelle* und *systemische* Faktoren bedingt sind. Um Verhalten zu verändern, ist es daher essenziell, dass auch systemische und individuelle Interventionsansätze verfolgt werden [8, 9]. Im Folgenden zeigen wir am Beispiel vom Ernährungsverhalten, wie *Behavioural and Cultural Insights *auf individueller und struktureller Ebene beitragen können, soziale Unterschiede im gesundheitsrelevanten Verhalten besser zu verstehen und zu verändern.

## Strukturelle Unterschiede: Ernährungsumgebungen

**Abb. 1:**
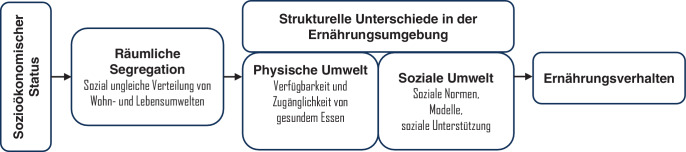
Ungleiche Verteilung von Ernährungsumgebungen. (Nach [13]; eigene Abbildung)

Ernährungsverhalten umfasst verschiedene Handlungen, die vor allem durch ihre wiederholte Ausübung die Gesundheit beeinflussen: Je mehr und je regelmäßiger Obst und Gemüse gegessen werden (zumindest bis 800 g/Tag), desto größer die protektiven Effekte auf die Mortalität [10]. Ähnlich sieht es mit gesundheitsschädlichen Nahrungsmitteln aus: Je mehr verarbeitete Fleischprodukte, wie z. B. Salami, konsumiert werden, desto höher der karzinogene Effekt [11]. Wiederholt und gewohnheitsmäßig ausgeübtes Verhalten wie Ernährung wird dabei stärker von Umgebungsfaktoren beeinflusst als einmalig ausgeübtes Verhalten [12]. Diese *Ernährungsumgebung* (Abb. 1) wird durch das soziokulturelle und politische Umfeld beeinflusst und umfasst Aspekte wie Verfügbarkeit, Erschwinglichkeit, Werbung und Nachhaltigkeit von Lebensmitteln. Ein einflussreiches Modell von Karen Glanz [13] unterscheidet 4 Dimensionen der Ernährungsumgebung: (a) Lebensmittelverkaufsstellen wie Restaurants, Imbissbuden und Supermärkte sowie deren Merkmale wie Öffnungszeiten, Entfernung und Dichte; (b) Alltagsumgebungen wie Zuhause, Arbeitsplatz oder Schule und deren Einfluss auf die Ernährung; (c) Konsumumgebungen, einschließlich der Verfügbarkeit gesunder Alternativen, Sonderangebote und Produktplatzierungen und (d) Informationsumgebungen wie Werbung und Medien.

Sowohl die subjektiv wahrgenommene Ernährungsumgebung als auch die über geografische Daten objektiv indizierte Nähe zu Geschäften, Restaurants oder Fast-Food-Verkaufsstellen hängen mit Ernährungsentscheidungen und Ernährungsverhalten zusammen [14]. So konnten in einer US-amerikanischen Studie [15] mit mehr als einer Million Teilnehmenden und routinemäßig über eine Fitness-App erhobenem Ernährungsverhalten gezeigt werden, dass Menschen, die besseren Zugang zu gesunder Ernährung hatten (hier: geringere Entfernung zu Supermärkten) und weniger Zugang zu Fast Food (hier: weniger Fast-Food-Restaurants), mehr Obst und Gemüse sowie weniger Fast Food konsumierten. Auch die Wahrnehmung sozialer Faktoren in der Umgebung kann einen Einfluss auf die Ernährung haben. Studien zeigen, dass soziale Normen – insbesondere die wahrgenommene Angemessenheit von Essen – je nach Situation variieren und dadurch Unterschiede im Snackkonsum erklären können [16]. Ernährungsumgebungen sind allerdings sozial stratifiziert. So finden sich in sozial benachteiligten Gebieten mehr Discounter, mehr Fast-Food-Ketten, weniger Bioläden und Restaurants sowie mehr Werbung für ungesunde Nahrungsmittel [17]. Soziale Unterschiede in Umgebungsfaktoren können so Ungleichheiten in ernährungsbezogenen Gesundheitsfolgen erklären [18]. Gleichzeitig wirkt sich die Ernährungsumgebung sozial ungleich auf Ernährung aus. In einer aktuellen Studie konnten wir zeigen, dass die wahrgenommene Verfügbarkeit von zuckerreichen Getränken bei Personen mit niedrigeren Bildungsabschlüssen stärker mit dem Konsum zusammenhängt als bei Personen mit höheren Bildungsabschlüssen [19]. Analog fand eine US-amerikanische Studie [15], dass soziale Unterschiede den Zusammenhang zwischen Zugang zu gesunder Ernährung und Konsum verändern – besonders in Gebieten mit überwiegend hispanischer oder schwarzer Bevölkerung.

## Individuelle Prozesse und soziale Unterschiede

Unterschiede im Ernährungsverhalten entlang sozialer Ungleichheitsdimensionen können auf unterschiedliche zugrunde liegende Mechanismen hinweisen: So kann beispielsweise der Bildungsstand beeinflussen, ob und wie Personen gesundheitliches Wissen erwerben und interpretieren, was wiederum zu Unterschieden im Ernährungsverhalten und Gesundheitszustand führen kann (z. B. [20]). Ein niedrigeres Einkommen kann hingegen eingeschränkten Zugang zu gesunden Lebensmitteln und mehr Alltagsstress bedeuten, was die Zeit für die Mahlzeitenzubereitung begrenzt (z. B. [21]) und so zu Unterschieden im Ernährungsverhalten führt. Die individuelle Migrationsgeschichte ist häufig mit gesünderen, auf kulturellen Unterschieden basierenden Ernährungsgewohnheiten verbunden; diese Unterschiede treten bei Migrant:innen der zweiten Generation jedoch seltener auf (z. B. [22]). Diese Dimensionen – Bildung, Einkommen, Migrationsgeschichte – sind nicht unabhängig voneinander. So haben z. B. Frauen mit Migrationsgeschichte in Deutschland meist geringere Einkommen als Frauen insgesamt oder auch Männer mit Migrationsgeschichte [23]. Dieses Phänomen wird als „Intersektionalität“ bezeichnet: Das Zusammenwirken verschiedener Dimensionen sozialer Ungleichheit führt zu spezifischen Erfahrungen von Diskriminierung und Benachteiligung, die insbesondere für Gesundheitsrisiken bedeutsam sind (z. B. [24]). Die quantitative Erfassung solcher intersektional benachteiligten Gruppen wurde erst in den letzten Jahren durch neuere Analysemethoden möglich. In entsprechend großen Datensätzen lassen sich z. B. durch Maschinenlerntechniken oder Entscheidungsbäume Gruppen erkennen, die durch spezifische Kombinationen von Dimensionen besonders benachteiligt sind. Entscheidungsbäume sind hierarchische Modelle, die Daten schrittweise in zunehmend homogene Untergruppen aufteilen, um relevante Muster für ein Zielkriterium zu identifizieren [25]. Werden soziale Dimensionen als Prädiktoren eingesetzt, werden die Merkmale ausgewählt, die den stärksten Zusammenhang mit dem Zielkriterium aufweisen. Dies ermöglicht die Identifizierung differenzierter, intersektionaler Subgruppen [26]. Moderne Maschinenlernverfahren erfassen dabei auch in komplexen Datenstrukturen nichtlineare Zusammenhänge und Wechselwirkungen zwischen verschiedenen sozialen Dimensionen, die mit konventionellen statistischen Methoden schwer zu erfassen wären.

So ausgewertet zeigen z. B. Daten des European Health Interview Survey für Deutschland, dass Geschlecht, Bildung, Erwerbsstatus und Migrationsgeschichte relevante Unterschiedsdimensionen für ungesunde Ernährung sind: Bei Männern mit hoher Bildung (Master oder mehr) und Migrationsgeschichte aß ein doppelt so hoher Anteil (15,18 %) die empfohlene Tagesmenge von 5 Portionen Obst und Gemüse als bei Männern mit hoher Bildung ohne Migrationsgeschichte (7,56 %) – aber bei beiden weniger als bei allen Frauen mit hoher Bildung (22,94 %; [27]).

Die Einflussgrößen für solche Unterschiede finden sich potenziell in Theorien des Gesundheitsverhaltens, allerdings werden dort nur wenige überprüfbare Annahmen zum Zusammenhang von Einflussgrößen und sozialem Status gemacht. Unser Team untersucht im Rahmen eines von der Deutschen Forschungsgemeinschaft (DFG) geförderten Projekts (UNICON (Understanding Nutrition Inequalities in Context: Momentary and Persistent Processes)) diese Zusammenhänge genauer und konnte beispielsweise zeigen, dass sozioökonomische Unterschiede die Ausprägung psychosozialer Determinanten wie Einstellungen oder Absichten beeinflussen [7, 28] und damit soziale Unterschiede bei der Ernährung *mediieren* (siehe unten). Gleichzeitig konnten wir zeigen, dass sich die Zusammenhänge zwischen individuellen Determinanten und gesundheitsrelevantem Verhalten zwischen sozialen Gruppen unterscheiden: Sie sind bei Personen mit höherem Bildungsstand oder solchen, die in weniger benachteiligten Gebieten wohnen [29], stärker ausgeprägt – werden also durch sozioökonomische Indikatoren *moderiert*.

## Individuelle und umweltbezogene Determinanten als Mediatoren sozialer Unterschiede im Verhalten

Theorien des Gesundheitsverhaltens beschreiben Verhalten als Ergebnis sozial erworbener Determinanten wie Einstellungen, Kontrollüberzeugungen, soziale Normen oder Risikowahrnehmungen. Diese Kognitionen werden durch das Beobachten oder Interagieren mit anderen – direkt oder über Medien – erworben und weiterentwickelt. Ein zentraler Punkt der meisten Theorien ist die Annahme, dass der Einfluss dieser Kognitionen durch Verhaltensintentionen oder -ziele vermittelt wird.

**Abb. 2:**
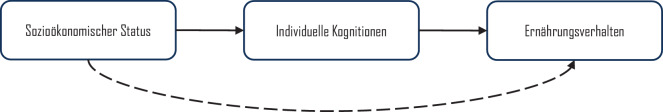
Mediationsannahme in sozialkognitiven Theorien des Gesundheitsverhaltens. (Eigene Abbildung)

Ein wichtiger, aber selten empirisch getesteter Aspekt ist Banduras Konzept der „triadischen reziproken Verursachung“ [30]. Demnach entsteht individuelles Verhalten aus der Wechselwirkung zwischen Umwelt, individuellen Kognitionen und dem Verhalten selbst. Dabei formt Verhalten die Umwelt, während Umweltbedingungen sowohl Verhalten als auch Kognitionen beeinflussen. Soziale Unterschiede im Gesundheitsverhalten können so durch sozial vermittelte Unterschiede in der Umwelt, persönlichen Erfahrungen und Interaktionen – und damit durch soziale Unterschiede in Überzeugungen über Konsequenzen, Kontrolle oder Normen – erklärbar sein. Das bedeutet: Unterschiede in individuellen Kognitionen (z. B. Einstellungen, Normen, Kontrollüberzeugungen) spiegeln soziale Unterschiede wider. Diese Kognitionen werden als vermittelnde Faktoren („Mediatoren“) zwischen sozialem Status und Gesundheitsverhalten angesehen (Abb. 2).

Einige Studien unterstützen diese Annahmen im Bereich Ernährung: Wir fanden beispielsweise in 2 repräsentativen internationalen Studien [7], dass ein höheres Bildungsniveau auf der einen und ein geringeres Einkommen auf der anderen Seite mit einem geringeren Verzehr von verarbeitetem Fleisch einherging. Einstellungen wirkten als partielle Mediatoren im Bildungszusammenhang: Desinteresse an Ernährung und eine Vorliebe für Fertigprodukte zeigten eine positive Assoziation, während die Präferenz für frische, regionale Produkte und gesundes Essen negativ assoziiert war. Der positive Zusammenhang zwischen Einkommen und verarbeitetem Fleischkonsum blieb hingegen unbeeinflusst. In einer Analyse europäischer Daten fanden wir zudem [28], dass Bildungseffekte auf gesunde Ernährung über geringeres Interesse an Fertigprodukten, aber auch höheres Vertrauen in Nahrungsmittelwerbung vermittelt waren, während sich Benachteiligung durch Alleinerziehendenstatus z. B. über geringer ausgeprägte Informationssuche auf gesunde Ernährung auswirkte. Neben solchen individuellen Mediatoren können auch umweltbezogene Faktoren eine gesunde Ernährung begünstigen, z. B. eine höhere wahrgenommene Verfügbarkeit gesunder Lebensmittel und Mahlzeiten, sozialer Zusammenhalt in der Gemeinschaft sowie ein starkes Sicherheitsgefühl und eine geringe Gewaltbelastung in der Umgebung. Eine Metaanalyse von Hagger et al. [31] legt allerdings nahe, dass solche mediierenden Effekte vor allem für geschlechts- und altersbedingte Unterschiede auftreten, aber bei bildungsbezogenen Gesundheitsverhaltensunterschieden weniger ausgeprägt sind.

Interventionen sollten daher gezielt soziale Unterschiede in Überzeugungen und Kognitionen adressieren, um gesundheitsrelevante Verhaltensweisen zwischen sozialen Gruppen anzugleichen.

## Moderation von Verhaltensdeterminanten durch sozioökonomische Faktoren

**Abb. 3:**
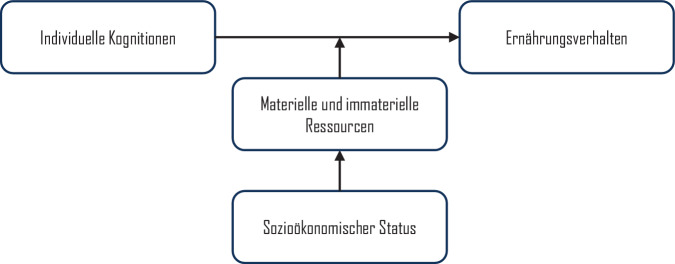
Moderation der Effekte individueller sozialer Kognition durch sozioökonomischen Status. (Eigene Abbildung)

Soziökonomische Faktoren können aber auch die Beziehung zwischen sozialkognitiven Variablen und Ernährungsverhalten *moderieren* – das heißt, das Ausmaß oder die Richtung des Einflusses von Intentionen, Selbstwirksamkeit oder Einstellungen auf das Ernährungsverhalten kann vom sozioökonomischen Status abhängen (Abb. 3).

Dabei können verschiedene Dimensionen des sozioökonomischen Status unterschiedliche Moderatoreffekte zeigen.

Zarnowiecki et al. [32] untersuchten bei Kindern den Zusammenhang zwischen Selbstwirksamkeit, Einstellungen zum Verzehr von Obst und Gemüse sowie gesundheitsfördernden Verhaltensweisen. Zusätzlich untersuchten sie, ob und wie sozioökonomische Merkmale der Mütter (z. B. Bildungsstand, Beruf, Beschäftigungsstatus und Haushaltseinkommen) diese Zusammenhänge beeinflussen. Die Ergebnisse zeigten geschlechtsspezifische Unterschiede. Bei Mädchen war der Zusammenhang zwischen Selbstwirksamkeit und gesundheitsförderndem Verhalten vom Beruf der Mutter abhängig. Bei Töchtern von Müttern in Arbeiterinnenberufen war dieser Zusammenhang schwächer als bei Mädchen, deren Mütter in akademischen oder angestellten Berufen tätig oder nicht erwerbstätig waren. Bei Jungen war der Zusammenhang zwischen ihren Einstellungen und dem tatsächlichen Obst- und Gemüseverzehr stärker, je höher das Haushaltseinkommen war. Sandvik et al. [33] fanden, dass der Beruf der Eltern die Zusammenhänge von Selbstwirksamkeit und der Intention, Obst zu essen, moderierte, wobei sich der stärkste positive Zusammenhang in den niedrigsten sozioökonomischen Schichten zeigte.

Die in den Studien beobachteten Moderationseffekte könnten darauf zurückzuführen sein, dass materielle und immaterielle Ressourcen ungleich verteilt sind. Diese Ressourcen beeinflussen, wie gut Menschen in der Lage sind, sozialkognitive Faktoren wie Intentionen oder Einstellungen in konkretes Verhalten umzusetzen. In sozial benachteiligten Gruppen können solche Faktoren – etwa Selbstwirksamkeit – kompensierend wirken, indem sie dabei helfen, strukturelle Barrieren zu überwinden. In privilegierteren Gruppen sind solche Barrieren seltener, weshalb diese Faktoren dort möglicherweise eine geringere Bedeutung haben. Die ungleiche Verteilung von Ressourcen spiegelt meist den sozioökonomischen Status wider. Materielle Einschränkungen, etwa ein geringes Einkommen, können es erschweren, regelmäßig gesunde Nahrungsmittel zu kaufen. Auch immaterielle (intangible) Ressourcen wie verfügbare Zeit, die durch Mehrfachbeschäftigung oder familiäre Verpflichtungen eingeschränkt sein kann, beeinflussen die Umsetzung gesundheitsförderlicher Verhalten im Alltag.

## Soziale Unterschiede in dualen Prozessmodellen des Ernährungsverhaltens

Aktuellere Modelle (z. B. [34]) postulieren, dass gesundheitsrelevantes Verhalten und damit auch Ernährungsverhalten aus 2 parallel operierenden Systemen resultieren. Meistens wird dabei ein System unterschieden, in dem deliberative (abwägende), zielorientierte und damit verarbeitungsintensive reflektive Prozesse ablaufen, und ein parallel operierendes nicht reflektives oder impulsives System, in dem nichtintentionales Verhalten auf Grundlage von hedonischen Impulsen, situativen Einflussgrößen und Affekt ausgelöst wird – also eher extern und kontextabhängig bedingt ist. In jeder Situation sind damit potenziell widersprüchliche Systemimpulse vorhanden, beispielsweise in einem Café ein hemmender Impuls auf Grundlage von Abwägungen (z. B. Vorsätzen zu gesunder Ernährung) und ein Impuls auf Grundlage situativer Einflüsse (z. B. Duft frisch gebackener Schokokekse). Welcher Impuls dann tatsächlich handlungsleitend ist, also darüber entscheidet, ob wir Schokokekse essen oder nicht, hängt von der Verfügbarkeit selbstregulativer Kompetenzen und Ressourcen und vom Verhalten anderer in diesem sozialen Kontext ab [16, 35, 36]. Wie oben (Ernährungsumgebung) beschrieben, sind die Verfügbarkeit und Präsenz von Hinweisreizen und das Verhalten von Personen, die Einfluss auf andere ausüben können, bei gesunder oder weniger gesunder Ernährung sozioökonomisch stratifiziert. Abhängig von sozioökonomischen Faktoren sind Menschen unterschiedlich vielen Hinweisreizen und qualitativ unterschiedlichen sozialen Kontexten ausgesetzt, was wiederum Essverhalten beeinflusst. Zum Beispiel finden sich zumindest in den USA in sozial benachteiligten Wohngebieten mehr Außenwerbung für ungesunde Lebensmittel oder zuckerhaltige Getränke [17] sowie mehr Fast-Food-Restaurants. Diese Ungleichheiten in der Lebensmittelumgebung stehen in Zusammenhang mit ungesünderen Ernährungsgewohnheiten, insbesondere bei Personen mit niedrigem sozioökonomischen Status [37].

## Implikationen für Interventionen zur Reduktion sozialer Unterschiede in Ernährung

Die oben vorgestellten Ansätze zum Verständnis sozialer Unterschiede im Ernährungsverhalten bieten verschiedene Ansatzpunkte zur Verbesserung der Ernährung und möglicherweise auch zur Verringerung gesundheitlicher Ungleichheiten. Dabei ist aber Vorsicht geboten: Wenn beispielsweise Bedarfsanalysen unzureichend durchgeführt oder Interventionskomponenten verwendet werden, die für ressourcenschwache Gruppen nicht wirksam sind, könnten Interventionen unbeabsichtigt gesundheitliche Ungleichheiten verstärken [38]. Idealerweise berücksichtigen solche Maßnahmen, dass soziale Unterschiede im Ernährungsverhalten durch komplexe Systeme bedingt sind, und kombinieren systemorientierte, soziale und individuelle Schwerpunkte [8, 9] auf mehreren Ebenen, indem z. B. sowohl Faktoren in der gebauten und sozialen Nahrungsmittelumgebung, Faktoren innerhalb der Person und Wechselwirkungen angesprochen werden. Im Folgenden zeigen wir auf Grundlage der oben beschriebenen Ansätze einige Beispiele für Interventionen.

### Interventionen zur Reduktion ernährungsbezogener Ungleichheiten auf Grundlage systemischer Faktoren

#### Verkaufsstellen.

Neben den oben erwähnten Studien finden sich in vielen weiteren Arbeiten Hinweise darauf, dass vor allem in Städten das Angebot von gesunden Nahrungsmitteln sozial stratifiziert ist, sich also beispielsweise in sozial benachteiligten Stadtteilen ein größeres Angebot ungesunder und ein geringeres Angebot gesunder Lebensmitteln findet [17, 37]. Die gezielte Ansiedlung oder Unterstützung von Verkaufsstellen mit Vollsortiment oder Märkten, ggf. kombiniert mit Regulationen für die Verkaufsstellendichte [39] oder Werbung [40] für Fast Food könnte dabei helfen, das Angebot an gesunden Lebensmitteln zu ergänzen und so die Ernährungsumgebung zu verbessern. Dabei können Supermärkte aber auch eine ambivalente Rolle spielen, weil Supermärkte mit Vollsortiment nicht nur gesunde Lebensmittel, sondern auch Junk Food anbieten [41].

#### Besseres Essen und Essensbedingungen in Gemeinschaftsverpflegung und Restaurants.

Mehr gesunde und ausgewogene Optionen in Einrichtungen der Gemeinschaftsverpflegung (Kantinen, Mensen, Cafeterias) und in Restaurants können an sich schon eine Verbesserung der organisationalen Nahrungsmittelumgebung (siehe oben) darstellen, können aber, wenn sie neben anderen Optionen im Sinne einer verbesserten Auswahlumgebung [9] prominenter angeboten werden, auch direkt individuelle Entscheidungen hin zu gesünderer Ernährung beeinflussen. Solche „Nudges“ werden oft als verhaltensändernde Optionen diskutiert, die wenig individuelle Entscheidungslatenz und Ressourcen benötigen und daher potenziell auch soziale Unterschiede in der Ernährung verringern könnten. In einem systematischen Review unter Berücksichtigung sozialer Ungleichheit [42] konnten wir zeigen, dass Nudges, die sich auf verhaltensbezogene Aspekte der Entscheidungsumgebung bezogen (z. B. Portionsgrößen), über soziale Gruppen hinweg effektiv waren. Allerdings waren Nudges, die auf Informationsvermittlung abzielten, eher bei sozial nicht benachteiligten Personen effektiv. Daneben kann aber auch gemeinsames Essen gesunde Ernährung fördern. Beispielsweise führte in Experimenten eine längere Mahlzeitdauer dazu, dass Kinder in der Schule mehr Obst und Gemüse aßen [43]; Kinder und Eltern im Familienkontext nahmen mit längerer Mahlzeitdauer mehr Obst und Gemüse (aber nicht mehr Dessert) zu sich [44].

#### Fiskalische Maßnahmen: Besteuerung von hochkalorischen und Subventionierung von gesunden Lebensmitteln.

Fiskalische Maßnahmen werden kontrovers diskutiert. Einerseits können regulatorische Eingriffe wie reduzierte Mehrwertsteuersätze für gesunde Lebensmittel die Ernährung allgemein verbessern. Andererseits gibt es Hinweise, dass sozial besser gestellte Personen, die sich ohnehin schon gesünder ernähren, stärker von solchen Maßnahmen profitieren als sozial benachteiligte Menschen [45]. Bei der Einführung einer zusätzlichen Steuer auf stark zuckerhaltige Getränke in Mexiko fanden sich größere Reduktionen im Konsum solcher Getränke in sozial benachteiligten Haushalten [46], die vorher eher mehr solcher Getränke konsumiert hatten. Allerdings lässt sich hier auch argumentieren, dass diese Effekte dadurch bedingt sein könnten, dass in diesen Haushalten die höheren Kosten besonders stark bemerkbar waren.

Bei den vorgestellten systemischen Ansätzen ist auch die Frage wichtig, ob und in welchem Umfang die regulatorischen Eingriffe, die zur Umsetzung solcher Maßnahmen notwendig sind, in der Bevölkerung als akzeptabel wahrgenommen werden. Eine aktuelle Studie in Deutschland [47] legt nahe, dass gut begründete strukturelle Maßnahmen zur Verbesserung der Ernährung durchaus auf Akzeptanz stoßen können. Wie sich diese jedoch im Hinblick auf die Hyperpolarisierung von Gesundheitsthemen der praktischen Umsetzung auswirken, ist eine offene Frage.

### Interventionen zur Reduktion sozialer Unterschiede im Ernährungsverhalten auf Grundlage individueller Prozesse

#### Stärkung der Selbstwirksamkeit.

Geringes Vertrauen in die eigenen Fähigkeiten kann zu ungesunden Ernährungsgewohnheiten führen [48]. Prestwich et al. [49] zeigten in einer Metaanalyse, dass bestimmte theoriebasierte Techniken (Behaviour Change Techniques) zur Verhaltensänderung die Selbstwirksamkeit in Ernährungsinterventionen wirksam stärken können. Wirksam waren insbesondere Maßnahmen, die zur Selbstbeobachtung und Selbstkontrolle des Ernährungsverhaltens anregen, Rückmeldungen über Fortschritte geben und soziale Unterstützung einbinden. Besonders effektiv war die Kombination dieser Ansätze mit Elementen des Stressmanagements, um die Selbstwirksamkeit zu fördern.

Um die ernährungsbezogene Selbstwirksamkeit zu fördern, müssen neue, gesunde Verhaltensweisen ausprobiert und positive Erfahrungen gemacht werden. Da sozial benachteiligte Menschen oftmals weniger Ressourcen haben, sind niedrigschwellige Ansätze nötig. Medien und digitale, kostenlose Anwendungen können dabei helfen [7]. Beispiele sind Apps, die Ernährungswissen vermitteln und neue Rezepte anbieten. Um wirksam zu sein, sollten solche Anwendungen evidenzbasierte Techniken der Verhaltensänderung beinhalten, wie Feedback zu Rezepten, eine Auflistung eigener Leistungen und die Anpassung des Schwierigkeitsgrads basierend auf bisherigen Erfolgen [49]. Allerdings zeigen aktuelle Befunde, dass digitale Interventionen soziale Unterschiede in ihrer Wirksamkeit aufweisen: Personen mit niedrigerem soziökonomischen Status [50] oder aus strukturell benachteiligten Regionen [51] profitieren oft weniger von digitalen Angeboten. Während der Einfluss von Alter und Geschlecht auf Nutzung und Wirksamkeit gut erforscht ist, bleiben andere Ungleichheitsdimensionen, wie Religion oder ethnische Zugehörigkeit, weitgehend unbeachtet [51].

#### Früh angesetzte Ernährungsbildung in Schulen.

Da Ernährungsgewohnheiten früh in der Kindheit entstehen und ein niedriges Bildungsniveau mit ungesunden Ernährungsgewohnheiten zusammenhängt, sollte Ernährungsbildung bereits in der Schule ansetzen. Das erlernte Ernährungswissen kann sich positiv auf das eigene spätere Ernährungsverhalten auswirken [52] und die in der Klassengemeinschaft gemachten positiven Erfahrungen können zur Entwicklung einer positiven Einstellung gegenüber Ernährung beitragen, die wiederum gesunde Ernährungsgewohnheiten fördert [35]. Niedrigschwellige Angebote für Eltern mit niedrigem Bildungsniveau können Ernährungswissen fördern, Gesundheitswahrnehmungen stärken [48] und so auch die gesunde Ernährung ihrer Kinder unterstützen. Dabei ist es wichtig, auch Unterschiede in kulturellen Hintergründen zu berücksichtigen, weil sie ernährungsbezogene Einstellungen und Gewohnheiten beeinflussen [53]. Das bedeutet gleichzeitig, dass Interventionen und Programme unterschiedliche kulturelle Einflüsse und Identitäten beachten sollten, um zusätzliche Benachteiligungen zu vermeiden.

## Schlussfolgerungen und Fazit

Soziale Unterschiede im Ernährungsverhalten tragen erheblich zu gesundheitlichen Ungleichheiten in Krankheitslast und Lebenserwartung bei. Während das Ausmaß dieser Ungleichheiten gut dokumentiert ist, sind die zugrunde liegenden Mechanismen und effektive Interventionsansätze unzureichend erforscht. Studien zeigen, dass sowohl strukturell-systemische als auch individuelle Faktoren eine zentrale Rolle spielen. Die Ernährungsumgebung ist sozial stratifiziert, was zu einer ungleichen Verfügbarkeit gesunder Lebensmittel führt und Ernährungsentscheidungen beeinflusst. Gleichzeitig moderieren sozioökonomische Faktoren den Einfluss individueller Determinanten wie Einstellungen oder Selbstwirksamkeit. Zur Verringerung ernährungsbedingter Ungleichheit sind Ansätze erforderlich, die *Behavioural and Cultural Insights* berücksichtigen und sowohl systemische als auch individuelle Interventionen umfassen. Regulierende Maßnahmen wie Werbeverbote oder fiskalische Anreize können die Ernährungsumgebung verbessern, während verhaltensbasierte Strategien, wie die Förderung von Selbstwirksamkeit oder frühzeitige Ernährungsbildung individuelle Handlungsspielräume erweitern und so zur Verringerung von Ungleichheiten beitragen können. Erfolgreiche Interventionen müssen jedoch sozial differenzierte Wirkmechanismen berücksichtigen, um unbeabsichtigte Verstärkungen bestehender Ungleichheiten zu vermeiden.
